# Prevalence of Small Intestinal Bacterial Overgrowth Syndrome in Patients with Non-Alcoholic Fatty Liver Disease/Non-Alcoholic Steatohepatitis: A Cross-Sectional Study

**DOI:** 10.3390/microorganisms11030723

**Published:** 2023-03-10

**Authors:** Paraskevas Gkolfakis, Georgios Tziatzios, Gabriela Leite, Ioannis S. Papanikolaou, Elias Xirouchakis, Ioannis G. Panayiotides, Athanasios Karageorgos, Maria Jesus Millan, Ruchi Mathur, Stacy Weitsman, George D. Dimitriadis, Evangelos J. Giamarellos-Bourboulis, Mark Pimentel, Konstantinos Triantafyllou

**Affiliations:** 1 Hepatogastroenterology Unit, Second Department of Internal Medicine-Propaedeutic, Medical School, National and Kapodistrian University of Athens, 12462 Athens, Greece; 2 Medically Associated Science and Technology (MAST) Program, Cedars-Sinai, Los Angeles, CA 90048, USA; 3 Department of Gastroenterology and Hepatology, Athens Medical Palaio Faliron General Hospital, 17562 Palaio Faliron, Greece; 4 2nd Department of Pathology, Medical School, National and Kapodistrian University of Athens, 124622 Athens, Greece; 5 4th Department of Internal Medicine, Medical School, National and Kapodistrian University of Athens, 12462 Athens, Greece

**Keywords:** non-alcoholic fatty liver disease, non-alcoholic steatohepatitis, small intestinal bacterial overgrowth, cirrhosis, microbiome

## Abstract

Introduction: Non-alcoholic fatty liver disease (NAFLD) is a multifactorial, wide-spectrum liver disorder. Small intestinal bacterial overgrowth (SIBO) is characterized by an increase in the number and/or type of colonic bacteria in the upper gastrointestinal tract. SIBO, through energy salvage and induction of inflammation, may be a pathophysiological factor for NAFLD development and progression. Aim/Methods: Consecutive patients with histological, biochemical, or radiological diagnosis of any stage of NAFLD (non-alcoholic fatty liver [NAFL], non-alcoholic steatohepatitis [NASH], cirrhosis) underwent upper gastrointestinal endoscopy. Duodenal fluid (2cc) was aspirated from the 3rd–4th part of duodenum into sterile containers. SIBO was defined as ≥10^3^ aerobic colony-forming units (CFU)/mL of duodenal aspirate and/or the presence of colonic-type bacteria. Patients without any liver disease undergoing gastroscopy due to gastroesophageal reflux disease (GERD) comprised the healthy control (HC) group. Concentrations (pg/mL) of tumor necrosis factor alpha (TNFα), interleukin (IL)-1β, and IL-6 were also measured in the duodenal fluid. The primary endpoint was to evaluate the prevalence of SIBO in NAFLD patients, while the comparison of SIBO prevalence among NAFLD patients and healthy controls was a secondary endpoint. Results: We enrolled 125 patients (51 NAFL, 27 NASH, 17 cirrhosis, and 30 HC) aged 54 ± 11.9 years and with a weight of 88.3 ± 19.6 kg (NAFLD vs. HC 90.7 ± 19.1 vs. 80.8 ± 19.6 kg, *p* = 0.02). Overall, SIBO was diagnosed in 23/125 (18.4%) patients, with Gram-negative bacteria being the predominant species (19/23; 82.6%). SIBO prevalence was higher in the NAFLD cohort compared to HC (22/95; 23.2% vs. 1/30; 3.3%, *p* = 0.014). Patients with NASH had higher SIBO prevalence (6/27; 22.2%) compared to NAFL individuals (8/51; 15.7%), but this difference did not reach statistical significance (*p* = 0.11). Patients with NASH-associated cirrhosis had a higher SIBO prevalence compared to patients with NAFL (8/17; 47.1% vs. 8/51; 15.7%, *p* = 0.02), while SIBO prevalence between patients with NASH-associated cirrhosis and NASH was not statistically different (8/17; 47.1% vs. 6/27; 22.2%, *p* = 0.11). Mean concentration of TNF-α, IL-1β, and IL-6 did not differ among the different groups. Conclusion: The prevalence of SIBO is significantly higher in a cohort of patients with NAFLD compared to healthy controls. Moreover, SIBO is more prevalent in patients with NASH-associated cirrhosis compared to patients with NAFL.

## 1. Introduction

Non-alcoholic fatty liver disease (NAFLD) remains the main cause of abnormal liver function tests in Western countries [1,2]. The definition of NAFLD requires the exclusion of ongoing or recent excessive alcohol consumption and other causes of liver steatosis [3]. Histologic evidence of liver fatty infiltration or, in the absence of histology, gamma-glutamyl transferase (γ-GT) and/or aminotransferase elevation, as well as compatible sonographic findings, are sufficient for diagnosis. Moreover, modern imaging modalities, such as controlled attenuation parameter (CAP) elastography and magnetic resonance proton density fat fraction, have been developed to quantify fatty infiltration and provide reliable evidence of NAFLD for diagnosis and clinical follow up [4]. The spectrum of the disease encompasses non-alcoholic fatty liver (NAFL), non-alcoholic steatohepatitis (NASH), NASH-related cirrhosis, and hepatocellular carcinoma [5]. Despite the fact that obesity, diabetes mellitus, and hypertriglyceridemia are the main risk factors for the development and progression of NAFLD [6], the pathogenetic factors that lead to NAFLD development have not been fully clarified. An emerging approach engaging liver steatosis with metabolic syndrome and their cumulative impact on cardiovascular diseases, has generated the new term of metabolic syndrome-associated fatty liver disease (MAFLD). However, this new nomenclature has not been universally accepted to replace NAFLD, due to the identified limitations on its applicability, especially regarding liver steatosis cases without metabolic syndrome [7]. More specifically, the historical “two hits” hypothesis regarding NAFLD pathogenesis [8] has been replaced by the “multiple parallel” hits hypothesis [9,10], where inflammation may either precede or follow simple steatosis with multiple factors, namely lipotoxicity, increased oxidative stress, mitochondrial dysfunction, and iron overload, acting in parallel to promote NASH [10]. This is also supported by studies where NAFLD is diagnosed in patients with chronic inflammatory conditions, such as inflammatory bowel diseases, even when their metabolic profile is normal, as indicated by the dominant term of sarcopenic NAFLD [11]. Moreover, a genetic predisposition to the disease development and progression cannot be excluded [12].

Interesting experimental data from the field of obesity have suggested a potential correlation between gut microbiota and NAFLD/NASH development [13,14,15,16]. This correlation could be mediated by a variety of mechanisms including direct action of the bacteria or their by-products (e.g., LPS-endotoxin) on hepatic parenchyma, bacterial translocation due to the increased intestinal permeability, and redundant salvage energy by altered gut microbiota [17,18]. Studies using fecal bacterial counts have demonstrated a clear correlation between the presence of NASH and a decreased percentage of Bacteroidetes, which represent one of the main bacterial phyla in the human gastrointestinal lumen [19,20,21,22]. In this regard, small intestinal bacterial overgrowth (SIBO), characterized by the abnormal presence of large numbers of colonic-type bacterial populations in the small intestine and normal microbiota imbalance, could be postulated to have a role in the pathogenesis of the disease, as the small intestine remains an important site for the functions of digestion and nutrient absorption. In this context, the present study aimed to evaluate the prevalence of SIBO in a cohort of patients with NAFLD, using a quantitative microbiological assessment of duodenal aspirate, a diagnostic modality that despite being considered the “gold standard” for SIBO diagnosis is not commonly used.

## 2. Patients and Methods

### 2.1. Study Design

This was a prospective, cross-sectional study conducted from April 2015 until March 2018. We designed this study according to the Strengthening the Reporting of Observational studies in Epidemiology (STROBE) guidelines (Supplementary Table S1) [23].

### 2.2. Study Population

#### 2.2.1. Non-Alcoholic Fatty Liver Disease Group

The NAFLD group consisted of patients visiting the outpatient hepatology clinic of the tertiary institutions ATTIKON University General Hospital and Athens Medical Palaio Faliron General Hospital with radiological and/or biochemical and/or histological evidence of fatty liver [3]. Patients with an age less than 18 years, use of antibiotics or probiotics within the last 3 months, ongoing alcohol consumption of any volume, presence of any other liver-related disease (e.g., viral hepatitis, metabolic liver disease, hepatocellular carcinoma), inability to provide informed consent, comorbidities (including stroke, chronic obstructive pulmonary disease), systemic diseases (e.g., systemic sclerosis), Crohn’s disease, chronic constipation (to exclude small intestinal methanogenic flora overgrowth [24]), previous gastrointestinal surgeries with postsurgical structural changes (ileocecal valve resection, gastric bypass, and Roux-en-Y), known jejunal diverticulum or stricture, chronic pancreatitis, or recent upper gastrointestinal bleeding or malignancies were considered exclusion criteria. Patient demographics, medical history, physical examination, and recent and ongoing medications were recorded at enrollment. Based on the baseline radiological and/or biochemical and/or histological assessment, each patient was classified either as non-alcoholic fatty liver (NAFL), non-alcoholic steatohepatitis (NASH), or as NASH-associated cirrhosis.

#### 2.2.2. Control Group

Individuals with negative medical history for comorbidities, no drug intake, no current or recent (within the last 3 months) use of proton pump inhibitors (PPI), or with H2 receptor antagonists undergoing upper gastrointestinal (GI) endoscopy for gastroesophageal reflux symptom evaluation were considered eligible for inclusion. Those finally having neither pathological findings after an endoscopy nor histological evidence of *Helicobacter pylori* (*H. pylori*) infection [25]—identified as non-erosive reflux disease patients—were enrolled in the control group.

### 2.3. Interventions

Enrolled study participants underwent upper GI endoscopy under conscious sedation with incremental doses of midazolam to exclude significant upper GI pathology and to test for *H. pylori* infection, after overnight fasting. No other specific recommendations regarding diet, smoking, or alcohol modification or cessation prior to endoscopy were given. In the case of significant pathological endoscopy findings, such as peptic ulcer disease, reflux esophagitis, Barrett’s esophagus, or malignancy, patients were excluded from the study. Prior to the procedure, an assistant using sterile gloves assembled the catheter and all other collecting devices and placed a sterile cap on the syringe in order to maintain aseptic conditions for all components of specimen collection and handling. For the next step, suction was disconnected to prevent cross-contamination by oral and upper gut flora. A sterilized upper endoscope, previously stored in a sterile place, was used by an endoscopist who also wore sterile gloves, to intubate the second/third portion of the duodenum with no air insufflation or water flushing. Upon entrance of the endoscope into the third section of the duodenum, the endoscopist passed the endoscopic retrograde cholangiopancreatography (ERCP) catheter through the biopsy channel of the endoscope and advanced it into the third and fourth portion of the duodenum for collection of the small bowel aspirate. At that time, gentle repeated suction with a sterile syringe connected to a 3-way stopcock was applied, allowing approximately 2–4 mL of duodenal fluid to be aspirated into a sterile container, as previously described in a standardized method [26]. Within approximately 2–5 min, the required amount of duodenal liquid was successfully aspirated, and the sterile containers were immediately transported to the laboratory.

### 2.4. Laboratory Investigation

Small bowel aspirates were quantitatively cultured under aerobic conditions, as in previous studies [26,27,28]. Briefly, one 0.1-mL aliquot with aspirate fluid was placed onto MacConkey agar (Becton Dickinson, Cockeysville, MD, USA), while another 0.1-mL aliquot of fluid was diluted 1:10 for four consecutive times into Mueller–Hinton broth and another 0.1 mL of each dilution was plated onto MacConkey agar. Plates were cultured for 24 h at 37 °C under aerobic conditions. The number of bacteria is expressed as colony-forming units (CFU)/mL by their log10 value, with a lower limit of detection of 10 CFU/mL. The study was focused on SIBO by aerobes since it has been suggested that third-part duodenum aspirates do not experience anaerobic conditions [26]. A colony count of ≥10^3^ CFU/mL in the duodenal culture was considered to be suggestive of the presence of SIBO [24]. In addition to the aforementioned diagnostic threshold for SIBO in the duodenal aspirate, results are also presented for diagnostic cutoffs of ≥10^4^ and ≥10^5^ CFU/mL. Commercial enzyme immunosorbent assays were used for the measurement of tumor necrosis factor (TNF) alpha, interleukin (IL)-1β (IL-1b), and IL-6 (Affymetrix Inc., Santa Clara, CA, USA), as previously described [29]. The lower limit of detection was 7.8 pg/mL for TNFα, 3.12 pg/mL for IL-6, and 2.34 for IL-1b. All measurements were performed by a technician blind to patient identity and clinical information.

### 2.5. Study Endpoints

The primary end point of the study was SIBO prevalence in a cohort of NAFLD patients based on the small bowel aspirate culture results. Secondary end points included the following: (i) comparison of SIBO prevalence between NAFLD and healthy controls; (ii) investigation of SIBO prevalence in the different stages of NAFLD (NAFL, NASH, and NASH-associated cirrhosis); (iii) comparison of SIBO prevalence between the different stages of NAFLD and healthy controls; and (iv) comparison of cytokine levels in duodenal fluid between NAFLD patients and healthy controls.

### 2.6. Statistical Analysis

Categorical data are presented as number and the prevalence as a percentage (%). Distribution of quantitative data was assessed for normality after Kolmogorov–Smirnov’s statistic and expressed as a mean (±SD or ±SEM) according to their distribution. Student’s *t* test was used for comparisons of variables with normal distribution, while we used non-parametrical tests to analyze categorical and non-continuous quantitative variables (chi-square statistic, Mann–Whitney U Test). Correlation of SIBO presence with other variables was assessed using logistic regression analysis. Any *p* value < 0.05 was considered significant for all statistical assessments.

### 2.7. Sample Size Estimation

Taking into account that SIBO prevalence is in NAFLD patients and that the general population is 25% and 5%, respectively, and in order to detect a difference at a significance level of 5% with a power of 80%, a G-power analysis determined that 30 patients should be recruited into the NAFLD group and 30 into the control group.

### 2.8. Ethical Approval

The protocol of this study was approved by the Attikon University General Hospital Ethics Committee (EBΔ 639/11-2-15) and is available at the Deutsches Register Klinischer Studien (DRKS)—German Clinical Trials Register, under registration number DRKS00009875. The study was conducted in accordance with the ethical principles of the Declaration of Helsinki and in compliance with good clinical practice guidelines. All participants were prospectively enrolled after ensuring them for anonymity and providing written informed consent. 

## 3. Results

Overall, we enrolled 125 patients: 95 in the NAFLD cohort and 30 healthy controls. Of them, 61 (48.8%) were female, and the mean age was 54.01 ± 11.95 years. Twenty-five patients (20%) suffered from diabetes mellitus type 2 and were on antidiabetic treatment and 19 (15%) used drugs that could affect the gastric pH (PPIs and/or H2-receptor antagonists). Regarding the NAFLD cohort, and based on the predefined clinical, laboratory, and radiological work-up, 51 (53.7%), 27 (28.4%), and 17 (17.9%) patients were classified as having NAFL, NASH, and NASH-associated cirrhosis, respectively. The baseline characteristics of the included patients are shown in Table 1.

### 3.1. Primary Endpoint

Based on the culture results, SIBO (prevalence [95% confidence interval (CI)]) was diagnosed in 22/95 (23.2% [14.7–31.6]) patients in the NAFLD cohort [male/female 16/6 (72.7%/27.3%); mean age (±SD) 58.2 (±11.3) years]. When applying a diagnostic threshold of >10^4^ CFU/mL of colonic-type aerobic bacteria in the duodenal aspirate for SIBO, the syndrome was diagnosed in 18/95 (18.9% [11.1–26.8]) patients, while with a diagnostic threshold of >10^5^ CFU/mL, SIBO was diagnosed in 11/95 (11.6% [5.1–18]) patients. *Escherichia coli* was the most common species identified in the culture of small bowel aspirate (10/22; 45.5%), followed by *Staphylococcus aureus* (4/22; 18.2%; Table 2).

### 3.2. Secondary Endpoints

**Figure 1:**
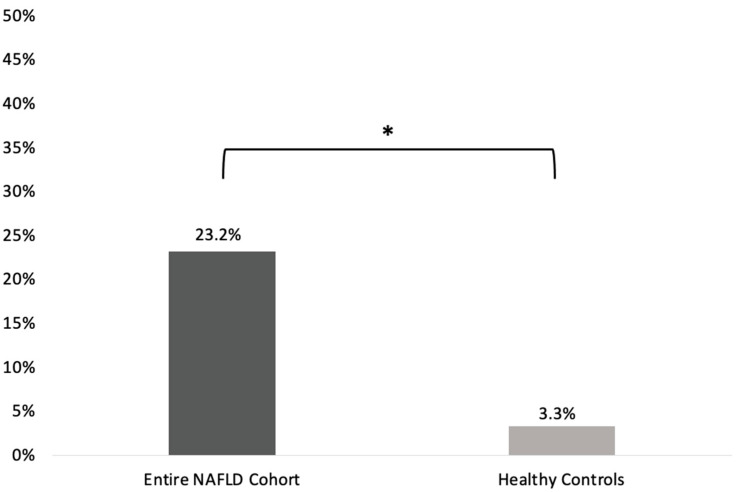
SIBO prevalence in the entire NAFLD cohort and the healthy controls group. SIBO: small intestinal bacterial overgrowth; NAFLD: non-alcoholic fatty liver disease. * denotes statistical significance.

**Figure 2:**
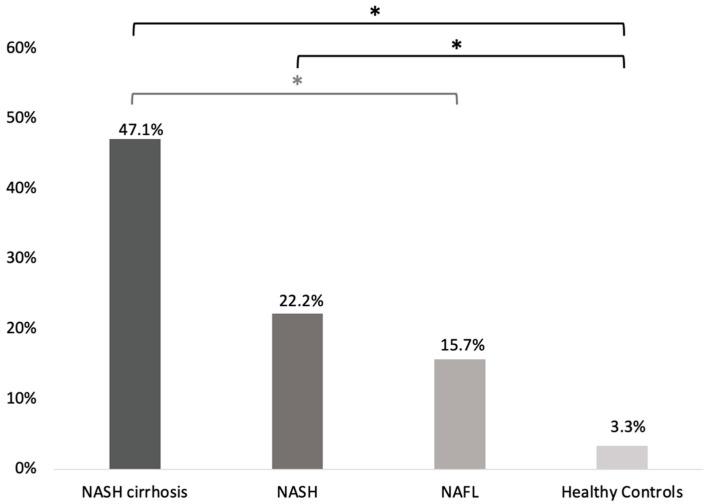
SIBO prevalence among patients with different stages of NAFLD and healthy controls. SIBO: small intestinal bacterial overgrowth; NAFL: non-alcoholic fatty liver; NASH: non-alcoholic steatohepatitis. * denotes statistical significance.

(i) SIBO prevalence was statistically significantly higher in the entire NAFLD cohort compared to the healthy controls group [22/95 (23.2%) vs. 1/30 (3.3%); *p* = 0.014; odds ratio (OR) (95%CI): 8.77 (1.12–66.7); Figure 1].
(ii) Regarding the different stages of the disease, SIBO prevalence was higher among patients with NASH-associated cirrhosis (8/17; 47.1% [23.3–70.8]) compared to patients with NAFL (8/51; 15.7% [5.7–25.7]; *p* = 0.02, OR (95%CI): 4.8 [1.4–16.1]) and patients with NASH (6/27; 22.2% [6.5–37.9]). However, the difference in SIBO preavalence between NASH-associated cirrhosis and NASH patients did not reach statistical significance (*p* = 0.11; OR (95%CI): 3.1 [0.8–11.6]). No statistically significant difference regarding SIBO prevalence was noted between patients with NASH and NAFL (*p* = 0.54; OR (95%CI): 1.5 [0.5–4.9]; Figure 2).
(iii) SIBO prevalence was significantly higher in NASH and NASH-associated cirrhosis patients compared to healthy controls (*p* = 0.04; OR (95%CI): 8.3 [1.01–74.1] and *p* < 0.001; OR (95%CI): 25.8 [2.8–234.8], respectively). Although numerically higher in NAFL, the difference in SIBO prevalence between NAFL patients and healthy controls did not reach significance (*p* = 0.14; OR (95%CI): 5.4 [0.6–45.4]; Figure 2).
(iv) Cytokine concentrations in the duodenal aspirates (mean pg/mL ± SEM) in NAFLD patients and healthy controls are presented in Table 3. TNFα concentrations in the duodenal aspirates did not differ between NAFLD subjects and the healthy control group (8.79 ± 0.86 vs. 7.80 ± 0.10, *p* = 0.533). NAFLD patients also had similar levels of IL-1β and IL-6 compared to healthy controls (7.63 ± 1.74 vs. 6.26 ± 2.76, *p* = 0.701 and 3.17 ± 0.04 vs. 3.14 ± 0.10, *p* = 0.648, respectively; Table 3). There were no differences in duodenal aspirate cytokine levels among the different stages of NAFLD (*p* ≤ 0.524).

Similar levels of TNFα, IL-1β, and IL-6 were also observed in those NAFLD patients who tested positive for SIBO compared to healthy controls (8.39 ± 0.56 vs. 7.8 ± 0.01, *p* = 0.240; 11.66 ± 5.55 vs. 6.26 ± 2.76, *p* = 0.362; and 3.12 ± 0.05 vs. 3.14 ± 0.10, *p* = 0.372, respectively). Finally, cytokine levels were also similar between NAFLD patients who tested negative for SIBO and those found to be positive for SIBO (8.39 ± 0.56 vs. 8.92 ± 1.12, *p* = 0.799; 11.66 ± 5.55 vs. 6.36 ± 1.48, *p* = 0.195; and 3.12 ± 0.02 vs. 3.18 ± 0.40, *p* = 0.439 for TNFα, IL-1β, and IL-6, respectively).

## 4. Discussion

Recently published data have suggested that SIBO could be implicated in the pathogenesis of NAFLD [17]. In the current study of a well-defined cohort of NAFLD patients, SIBO was diagnosed in almost one-fourth of them using quantitative culture of the proximal small intestine, which is considered the gold standard for SIBO diagnosis. Moreover, NAFLD patients had a significantly higher prevalence of SIBO compared to healthy controls, while SIBO was more frequent in patients with NASH cirrhosis compared to NAFL.

Although the definition of a clear pathophysiological relationship between SIBO and NAFLD remains an open question, a recent systematic review and meta-analysis demonstrated that patients with NAFLD are prone to suffering from SIBO at the same time [OR (95%CI): 3.82 (1.93–7.59)] [30]. However, the exact prevalence of SIBO among NAFLD patients varies in the literature, potentially due to significant variability in the criteria and methods used to define and diagnose both NAFLD and SIBO [31]. In this context, our results are in line with the results of the study by Guimaraes et al., who reported that the prevalence of SIBO among histologically confirmed NAFLD patients using the lactulose breath test as a diagnostic modality was 26.2% [32]. Different reports in the literature, both in NAFLD [33,34,35] and in NASH [36] patients that used either cultures of jejunal aspirates [33,34,36] or a lactulose breath test [35] to diagnose SIBO, reported an even higher prevalence of the syndrome, highlighting its frequent occurrence in this population. Specifically, SIBO prevalence was reported to be 40% among NASH individuals [36], while it approached or exceeded 35% in those with NAFL [33,34,35]. Moreover, among individuals with biopsy-proven NASH, the prevalence of SIBO was >43% both in those with concomitant metabolic syndrome as well as in those without [37]. Interestingly, in two of the studies that used the 10^5^ cfu/mL threshold after jejunal aspirate culture to diagnose SIBO, the prevalence of SIBO was reported to be 37.5% and 47.2% [33,34]. Finally, it is worth mentioning that the prevalence of SIBO in the control group was similar to those reported in the literature in studies performed either in the field of NAFLD [38] or other diseases [39]. 

Our study showed that the prevalence of SIBO among patients with NASH-associated cirrhosis was significantly higher compared with patients with NAFL. The relationship between SIBO prevalence and disease stage has been the focus of many studies in this field [33,34,35,38,40,41,42], with inconclusive results. One of the first studies, that included 136 patients with available liver biopsy who underwent a glucose hydrogen breath test for SIBO detection, demonstrated that SIBO was more frequent among patients with non-severe hepatic steatosis (26.3% vs. 10.3%, *p* = 0.127), whereas it did not differ in patients with NASH [41]. Moreover, in their study of 35 liver biopsy-proven NAFLD patients, Miele et al. concluded that the prevalence of SIBO increased significantly with the severity of steatosis (*p* = 0.001), without a significant correlation between SIBO and the presence of NASH (*p* = 0.84) [40]. Similar to our results, in a study by Scarpellini et al., a higher prevalence of SIBO, diagnosed using the lactulose breath test, was observed in patients with cirrhosis compared with non-cirrhotic patients (41.2% vs. 13.1%; *p* < 0.05), but no association was observed between SIBO prevalence and the grade of liver steatosis, as diagnosed using abdominal ultrasound [38]. On the other hand, a study by Shi et al. reported that as the severity of steatosis, expressed as the controlled attenuation parameter, increased, the prevalence of SIBO also increased, ranging from 32.6% to 88% for mild and severe steatosis, respectively [35]. Additionally, of interest, a recent study by Mikolasevic et al. that used jejunal aspirate culture to diagnose SIBO, reported that the prevalence of SIBO was higher among patients with biopsy-proven NASH compared with patients with NAFLD, but provided no evidence of NASH in the liver biopsy (68.1% vs. 29.1%; *p* < 0.001) [33]. However, in two other studies using either jejunal aspirate [34] culture or the glucose hydrogen breath test [42], SIBO prevalence did not differ by the histological stage of the disease [34] or according to the presence of severe steatosis [42]. More precisely, Fitriakusumah et al. did not find any significant association between SIBO and NAFLD development, while SIBO was also not associated with significant hepatic steatosis [42].

Despite these conflicting results, two points should be highlighted regarding the higher prevalence of SIBO with advanced stages of NAFLD, and especially with NASH-associated cirrhosis. First, patients with cirrhosis often have impaired intestinal motility that could lead to an increased orocecal transit time that could promote bacterial overgrowth, including in the small intestine, due to decreased intestinal clearance [35,43]. Second, proton pump inhibitor (PPI) intake can lead to a moderately increased risk of SIBO development as demonstrated in a meta-analysis of 7055 patients (OR [95%CI]: 1.71[1.20–2.43]) [44]. At the same time, proton pump inhibitor consumption is a frequent phenomenon among cirrhotic patients [45]. Thus, one cannot preclude an association between increased PPI intake and higher SIBO prevalence among NASH-related cirrhotic patients. However, in our cohort, PPI consumption did not differ among patients with different stages of the disease.

Accumulating evidence from published studies suggests a multifactorial contribution of SIBO in the development and progression of NAFLD [17,18,30,31,32,33,34,35,36,37,38]. The main proposed mechanisms include a disturbance of energy homeostasis leading to energy salvage, increased oxidative stress, enhancement of insulin resistance, increased ethanol production, and alterations in the metabolism of choline and bile acids [18,31,46]. Nucleotide-binding oligomerisation domain-like receptor protein NLRP3 and NLRP6, which belong to the family of inflammasomes, balance the immune response with the expression of interleukin IL-1b and IL-18, serving as regulators of normal microbiota and ameliorating NAFLD, whereas dysbiosis has been associated with a decrease in those inflammasomes and NAFLD occurrence in animal models [47,48,49]. Portal circulation and the liver–gut axis can also comprise the pathways of bacterial translocation to the liver, accompanied by pathogen-associated molecular patterns (PAMPs), danger-associated molecular patterns (DAMPs), which are recognized triggering factors of chronic inflammatory responses [50]. Moreover, increased permeability due to tight cell junctions disruption found in SIBO patients can lead to the translocation of bacteria and their products, such as endotoxins, while endotoxemia further induces CD14 mRNA and nuclear factor kappa B (NF-kB) expression that consequently activates Toll-like receptor 4 (TLR-4) and stimulates the production of proinflammatory cytokines such as tumor necrosis factor α (TNF-α), IL-1β, IL-6, and IL-8 [31,34,35,46]. Overproduction of these cytokines, but also the direct effect of bacterial products, such as lipopolysaccharides, contribute to the development of inflammation and insulin resistance and may be essential in the pathogenesis of NASH, liver fibrosis, and hepatocellular carcinoma [34]. In order to move a step beyond systematic responses with the aim of elaborating upon the potential role of mucosal immunity of the GI compartment on disease development and progression, we assessed cytokine levels (TNFα, IL-1β, and IL-6) in the duodenal aspirate of patients with NAFLD as well as in healthy controls. However, our analysis did not reveal any differences in any of the cytokine levels in duodenal samples among NAFLD subjects and healthy controls or between individuals with different stages of the disease. In any case, further confirmation of these findings in future trials is warranted.

Regarding the composition of the intestinal microbiota of NAFLD subjects, this has been the focus of different studies that have used culture-independent techniques, such as quantitative real-time polymerase chain reaction (qRT-PCR) and sequencing of the bacterial 16S ribosomal RNA (16S rRNA) gene, to assess, mainly, stool samples. One of the main outcomes of these studies was to evaluate the abundance of different phyla, particularly the Firmicutes/Bacteroidetes ratio, an indirect index of dysbiosis. While a lower abundance of Firmicutes was demonstrated in the feces of both children and adults with NASH compared to their healthy comparators [19,21], other studies did not demonstrate any alteration in the Firmicutes/Bacteroidetes ratio among NAFLD patients and healthy controls [20,22]. However, while the use of stool samples may underestimate or even cloak the contribution of small intestinal flora and small intestinal bacterial overgrowth in NAFLD pathophysiology [51], data on the duodenal microbiome composition of both NAFLD and SIBO patients are lacking. In a recently published study, Leite et al. used 16-s RNA sequencing to characterize the duodenal microbiome of patients diagnosed with SIBO by using duodenal aspirates [52]. The authors showed that duodenal aspirates from SIBO subjects had 4 × 10^3^-fold higher bacterial counts on MacConkey agar than duodenal aspirates from non-SIBO subjects (*p* < 0.0001) [52]. Moreover, SIBO subjects presented with decreased α-diversity, with a significant strong inverse correlation between the Proteobacteria and Firmicutes phyla [52]. In our study, we did not use culture-independent techniques to describe the duodenal microbiome, but it should be noted that the vast majority of the isolated bacteria in NAFLD patients with SIBO (72.6%) belonged to the Proteobacteria phylum, followed by Firmicutes (27.4%). In any case, direct application of advanced, culture-independent techniques on duodenal aspirates could reveal a likely site-dependent interaction between NAFLD and gut microbiota.

Duodenal fluid aspiration, although a broadly applied and acceptable technique, reflects the microbiota composition of a minor part of the small bowel. Bacterial diversity and phyla that could determine SIBO development and its impact on diseases, such as NAFLD, may be not isolated in all duodenal aspirate cultures, thus requiring jejunal or ileal fluid. However, the interventional nature of enteroscopic techniques, with the associated risks, does not allow spiral or balloon enteroscopy for the indication of fluid aspiration and culture. Emerging studies have assessed the role of dedicated devices to collect samples from the small bowel for microbiota analysis. More specifically, a simple low-cost 3D capsule and a robot capsule have been designed to obtain intestinal fluid or tissue, respectively, with the aim to provide a representative mapping of bowel microbiota and diagnose SIBO [53,54]. These promising technologies, needing clinical evaluation, could provide detailed information about the role and the identity of bacterial species in SIBO, with regard to NAFLD incidence and severity.

We should acknowledge a number of strengths related to our study. First, we used cultures of small bowel aspirates to diagnose SIBO, with a cut-off of 10^3^ cfu/mL, which is the suggested threshold for SIBO diagnosis in the literature [24]. This technique may be time-consuming, more expensive, and invasive and difficult for the patient requiring sedation, but it is now considered as the gold standard to make a SIBO diagnosis [24,55]. Second, the study design allowed us to enroll a large number of individuals, overcoming the initially estimated overall sample size of 60 subjects. Finally, all participants underwent a detailed laboratory and clinical work-up, allowing us to exclude any other liver-related pathologies and get a well-defined cohort of NAFLD patients. On the other hand, our study also has limitations that merit consideration. First, the cross-sectional design of the study precluded the establishment of a causal relationship between SIBO and NAFLD. A second limitation is that nonerosive reflux disease patients were enrolled in the control group. We acknowledge the fact that these patients are not typically healthy subjects, but it would be unethical to enroll patients to undergo a non-indicated endoscopy given the existing, albeit low, risk of complications. Third, we did not perform cultures for anaerobic organisms and deep sequencing analysis; however, this should be taken into consideration by future studies. Finally, liver biopsy, as an invasive modality with considerable complication rates, was not considered mandatory to enroll patients for ethical reasons and, as expected in a real life study, it was performed, as recommended [56], if there was uncertainty regarding the cause of liver injury or the stage of liver fibrosis.

## 5. Conclusions

To conclude, this prospective cross-sectional study in a well-defined cohort of NAFLD patients demonstrated that the prevalence of SIBO, using duodenal aspirate culture, is significantly higher among patients with NAFLD compared to healthy controls. In addition, SIBO is more prevalent among patients with NASH-associated cirrhosis compared to NAFL or NASH individuals. Further data are warranted in order to better explain a potential causal relationship.

## Figures and Tables

**Table 1:** Baseline patient characteristics.

	Entire NAFLD Cohort (n = 95)	NAFL (n = 51)	NASH (n = 27)	NASH Cirrhosis (n = 17)	Healthy Controls (n = 30)	* *p*-Value	** *p*-Value
Age (years), mean (±SD)	55 (10.9)	54.7 (9.2)	49.8 (11.8)	63.9 (8.5)	50.9 (14.6)	0.103	<0.001
Gender, n (%)						0.209	0.110
Female	43 (45.3)	27 (52.9)	12 (44.4)	4 (23.5)	18 (60)		
Male	52 (54.7)	24 (47.1)	15 (55.6)	13 (76.5)	12 (40)		
Weight (kg), mean (±SD)	90.7 (19.1)	89.2 (16.6)	91.1 (21.1)	94.4 (23.2)	80.8 (19.5)	0.016	0.630
Height (cm), mean (±SD)	176 (6.3)	180 (8.6)	171 (7.4)	171 (11.5)	171 (6.4)	0.644	0.786
Diabetes mellitus type 2, (%)						0.295	0.391
Yes	21 (22.1)	14 (27.5)	4 (14.8)	3 (17.6)	4 (13.3)		
No	74 (77.9)	37 (72.5)	23 (85.2)	14 (82.4)	26 (86.7)		
PPI and/or H2RA intake, n (%)						0.007	0.398
Yes	19 (20)	12 (23.5)	3 (11.1)	4 (23.5)	0 (0)		
No	76 (80)	39 (76.5)	24 (88.9)	13 (76.5)	30 (100)		

NAFLD: non-alcoholic fatty liver disease; NAFL: non-alcoholic fatty liver; NASH: non-alcoholic steatohepatitis; SD: standard deviation; PPI: proton pump inhibitor; H2RA: H2 receptor antagonist. * refers to differences between the entire NAFLD cohort and the healthy controls group. ** refers to differences between the different stages of the NAFLD cohort (NAFL, NASH, and NASH-associated cirrhosis).

**Table 2:** Bacterial species from duodenal aspirates of study participants with small intestinal bacterial overgrowth (SIBO).

Bacterial Species, n (%)	Entire NAFLD Cohort, n (%)	Healthy Controls, n (%)
*Escherichia coli*	10 (45.5)	-
*Staphylococcus aureus*	4 (18.2)	-
*Pseudomonas aeruginosa*	3 (13.6)	-
*Enterococus faecalis*	2 (9.1)	-
*Klebsiella pneumoniae*	1 (4.5)	-
*Providencia alcalifaciens*	1 (4.5)	-
*Acinetobacter* spp.	1 (4.5)	-
*Enterobacter cloacae*	-	1 (100)
Total, n/N (%)	22/95 (23.2%)	1/30 (3.3%)

NAFLD: non-alcoholic fatty liver disease.

**Table 3:** TNFα, IL-1b, and IL-6 concentrations in duodenal fluid from the NAFLD cohort and healthy controls.

Concentration in Duodenal Fluid *	Entire NAFLD Cohort (n = 95)	NAFL (n = 51)	NASH (n = 27)	NASH-Cirrhosis (n = 17)	Healthy Controls (n = 30)	*p*-Value **	*p*-Value ***
TNFα	8.79 ± 0.86	7.80 ± 0.10	10.69 ± 2.89	8.57 ± 0.72	7.80 ± 0.10	0.533	0.345
IL-1β	7.63 ± 1.74	8.51 ± 2.92	8.69 ± 2.75	3.45 ± 1.11	6.26 ± 2.76	0.701	0.524
IL-6	3.17 ± 0.04	3.22 ± 0.07	3.12 ± 0.04	3.12 ± 0.04	3.14 ± 0.10	0.648	0.417

* Measured and presented as mean (pg/mL) ± standard error of mean (SEM); ** difference between the entire NAFLD cohort and the healthy controls; *** difference among the different stages of the NAFLD cohort. NAFLD, non-alcoholic fatty liver disease; NAFL, non-alcoholic fatty liver; NASH, non-alcoholic steatohepatitis; IL, interleukin; TNF, tumor necrosis factor.

## Data Availability

The data presented in this study are available on request from the corresponding author. The data are not publicly available due to.

## Supplementary Materials

The following supporting information can be downloaded at: https://www.mdpi.com/article/10.3390/microorganisms11030723/s1, Table S1: STROBE Statement—checklist of items that should be included in reports of observational studies.
